# People with long-term conditions sharing personal health data via digital health technologies: A scoping review to inform design

**DOI:** 10.1371/journal.pdig.0000264

**Published:** 2023-05-24

**Authors:** Amy Rathbone, Simone Stumpf, Caroline Claisse, Elizabeth Sillence, Lynne Coventry, Richard D. Brown, Abigail C. Durrant

**Affiliations:** 1 Open Lab, School of Computing, Newcastle University, Newcastle upon Tyne, United Kingdom; 2 School of Computing Science, University of Glasgow, Glasgow, United Kingdom; 3 Department of Psychology, Northumbria University, Newcastle upon Tyne, United Kingdom; 4 School of Design and Informatics, Abertay University, Dundee, United Kingdom; University of Technology Sydney, AUSTRALIA

## Abstract

The use of digital technology amongst people living with a range of long-term health conditions to support self-management has increased dramatically. More recently, digital health technologies to share and exchange personal health data with others have been investigated. Sharing personal health data with others is not without its risks: sharing data creates threats to the privacy and security of personal data and plays a role in trust, adoption and continued use of digital health technology. Our work aims to inform the design of these digital health technologies by investigating the reported intentions of sharing health data with others, the associated user experiences when using these digital health technologies and the trust, identity, privacy and security (TIPS) considerations for designing digital health technologies that support the trusted sharing of personal health data to support the self-management of long-term health conditions. To address these aims, we conducted a scoping review, analysing over 12,000 papers in the area of digital health technologies. We conducted a reflexive thematic analysis of 17 papers that described digital health technologies that support sharing of personal health data, and extracted design implications that could enhance the future development of trusted, private and secure digital health technologies.

## Author summary

Many patients now use computers and smartphones to help themselves when they are living with a long-term condition. Recent technologies allow patients to share their own data with others but this can be risky. We review published work to investigate the user experiences that people had with technologies that allowed sharing data and how we can design better technology for sharing data with others that is trusted, private and secure.

## 1 Introduction

The use of digital technology amongst people living with a range of *long-term health conditions* (LTHCs) to support self-management [1–3] has increased dramatically. Online forums and social networking sites in which people living with LTHCs can share their experiences and expertise with their peers–other people living with the same condition–have been extensively investigated in terms of the informational, emotional and social support they can offer [3–8]. More recently, digital health technologies to *share and exchange health data* have been investigated, either through electronic patient health records (ePHRs) between patient and healthcare professionals [9–11], or through *personally generated health data* shared with others [11,12]. Personally generated health data is tracked through patients’ own devices, such as mobile apps, fitness trackers and other wearables, mostly through manual entry but increasingly tracking can be automated. This data is often obtained in the context of tracking and storing medication adherence, symptoms, side effects, and other activities and experiences, in a variety of ways [13–16] as part of individuals’ self-management of their condition(s). Thus, this includes data on patients’ physical activities such as steps and hours of sleep, emotional states such as mood and medical history such as symptoms they experienced. With the advent of novel wearable technology, it might also include more complex data such as heart rate and sleep patterns.

Sharing health data with others is not without its risks: sharing data is found to pose threats to the privacy and security of personal data [17,18] and plays a role in trust, adoption and continued use of digital health technologies [8–11,19,20]. We employ concepts common in *trust*, *identity*, *privacy and security (TIPS)* research. To share data with others, there needs to be some *trust*. We view trust as a social tie between two parties, in which one opens themselves up to vulnerability by sharing something with the other, with the expectation that the other party will behave appropriately [21]. Users will have a digital *identity* that provides access to and use of digital health technology [22] in order to authenticate themselves and interact with systems, services and processes, either in the physical or digital world. Digital identities comprise and store one or more attributes, such as personal data, or medical records. *Privacy* and *security* are important aspects for trust, where privacy concerns a person’s ability to choose how their data are revealed to others [23,24], while security refers to the safety of a person’s data and protection against unwanted access [13,23]. Significant challenges remain for digital health technology designers to implement private, secure and trusted systems.

A number of related scoping reviews have been conducted in recent years, about: adoption of ePHRs by physicians and patients observing barriers to patients for involvement in their health management [25]; factors that impact patients’ use of ePHRs [26,27]; elements of user trust in digital health systems–pre-pandemic [28]; and digital literacy during COVID ‘Infodemic’ shaping digital health technology use [29]. None of these reviews, to date, has focused on how digital health technologies afford the trusted sharing of personally generated health data for the self-management of health, focusing on LTHCs and incorporating design evaluations.

Our aim with this review is to inform the design of these digital health technologies by considering current best practice. We set out to investigate the reported intentions of sharing personally generated health data with others and the associated user experiences which drive adoption and continued use of digital health technologies. The second aim of our work was to highlight TIPS considerations for designing digital health technologies that support the trusted sharing of personally generated health data to support self-management of LTHCs. Our motivating research questions follow.

1. Why do users share personally generated health data via digital health technologies?
2. What are user experiences (UX) of sharing personally generated health data via digital health technologies, especially relating to trust, identity, privacy and security affecting the sharing of personally generated health data via digital health technologies?
3. What are the design implications for digital health technologies that support the trusted sharing of personally generated health data for self-management of long-term health conditions?

To address these research questions, we conducted a scoping review [30] in this area. We analysed over 12,000 papers in the area of digital health technologies, then we conducted a thematic analysis of 17 papers that described digital health technologies that support sharing of personally generated health data in some form, and extracted design implications that could enhance the development of trusted, private and secure digital health technologies in future.

The review is organised as follows. We first present our methods for conducting our scoping review following the PRISMA-ScR process, giving details about the eligibility criteria, information sources, search terms, selection strategy and data charting and synthesis. We then present the results of conducting the scoping review, including an overview of the characteristics of the included publications and the results of a thematic analysis. We then discuss the results with a view to future research directions and conclude with a summary of our work.

## 2 Methods

We adopted a scoping review using the PRISMA-ScR (Preferred Reporting Items for Systematic Reviews and Meta-Analyses extension for Scoping Reviews) extension [30]. The checklist consists of 20 essential and two optional reporting items. The method was supported by adopting a critical-reflective approach within the research team, to account for the interdisciplinary nature of the subject matter and the speed of digital health technology advances. Our team itself was comprised of experts in Health Psychology, Interaction Design and Computer Science, working together in the interdisciplinary field of Human Computer Interaction (HCI); and we adopted an interpretative stance to reflect on how the differing disciplinary perspectives of paper authors and researchers may shape their (and our, within the review team) choice of methodology, and use of language, terminology in study protocols, analyses, and reports of work. Our critical-reflective approach to the review was facilitated via regular meetings wherein the team discussed and reflected upon all steps of the scoping review method. This meant that we necessarily followed an *iterative* review process within each step: a single researcher (the first author and lead researcher) initially conducted the work in each step very quickly, the results of which were then brought back to the team for discussion, and then any refinements or changes were reintegrated into an enhanced cycle of work. In some cases, we iterated through this process multiple times. For example, concerning search terms, the lead researcher conducted the initial review based on a selection of search terms, the results of which were then discussed in a team setting. Based on this discussion we added synonyms and adjusted the search strategy. These refined terms were then implemented by the lead researcher. We iteratively cycled through this process three times before establishing the final search terms. We took similar approaches to eligibility criteria, information sources, selection strategy and especially data charting and synthesis.

### 2.1 Eligibility criteria

To be eligible for inclusion within the review, publications had to be in English but we did not place any geographic restrictions on the publication. We restricted the time range of publications from 2008 to February 2022, accounting for the rise in mobile applications (apps) popular in digital health technologies in that time period [31]. Included publications had to conduct studies or evaluations with individuals with LTHCs sharing personally generated health data via digital health technologies with healthcare professionals or others. We excluded any data sharing between systems and different types of technology. Studies were excluded if they did *not* include a user evaluation or study. This was to ensure that conclusions drawn from the review were based on real-world, empirical evidence, from an end user perspective. Both quantitative and qualitative studies were eligible for inclusion.

### 2.2 Information sources

We conducted a comprehensive search of literature in applicable, online, bibliographic databases. We included APA PsychNet including PsychInfo, PsychArticles, PsychBooks & PsychExtra, ProQuest, Sage, Web of Science, PubMed, PubMed Central, ACM Digital Library, Science Direct, IEEE Xplore and Scopus.

### 2.3 Search terms

Through our critical-reflective approach we agreed on a set of search terms to retrieve appropriate publications. The key search terms were “digital health technology”, “personal health data” and “sharing”. For these search terms we also used relevant synonyms, for example, “mHealth” (i.e. digital health technologies over mobile devices), “patient-generated data” and “self-tracked data”. We also included search terms to indicate who these data might be shared with i.e., “peers” or “community”, and focused on “self-management” applications rather than clinical systems. Where hyphens were present in search terms, the search was repeated without to ensure that no relevant publications were missed. Boolean terms (AND, OR, NOT) were initially to be utilised during the search but we settled on using the more restrictive “AND” conjunction to focus our search. Searches of these terms were conducted over the full text of the publication, also covering the Abstract and keywords. Applying these search terms to our 10 information sources yielded a total of 12,178 records.

### 2.4 Selection strategy

Following the previous step, we deliberated on eligibility criteria for inclusion in the review, again guided by our critical-reflective approach. At this stage we removed duplicate publications that were returned from different databases, plus any abstracts, books or theses. A title and abstract review was conducted and publications that did not satisfy the eligibility criteria were discarded from further analyses. We then screened the remaining full text of publications for relevance. Publications were excluded for the following reasons: non-empirical studies, studies that did not include the use of digital health technologies, involving users not experiencing LTHCs, no investigations relevant to TIPS (we were guided by the definition of TIPS terms given in the Introduction), or no first-hand reports of their experience from users.

### 2.5 Data charting and synthesis

Information from publications was extracted using a standardised format to characterise the included work. The information covered publication metadata, information such as, sample size, study type (quantitative/qualitative), age range, population, and study methods. We describe the findings from this analysis in Results.

We then conducted a reflexive thematic analysis [32,33] on the included publications. We started by employing a deductive approach to draw out themes around the topics of intention, user experience, and design features related to TIPS, consistent with our research aims. We familiarised ourselves with the data and then coded the data inductively (lead researcher supported by team with members coordinating individual coding tasks for collaborative work) to generate initial themes from the codes and coded data. Coding was reviewed and themes were further defined, named and refined into sub-themes if necessary. All researchers were involved to ensure reliability and validity and reduced the risk of bias. Multiple rounds of discussion within the team took place to finalise coding. We applied these codes to the text of each publication. Each code could be applied multiple times in each publication and codes are not mutually exclusive; different codes can be applied to the same publication. The finalised codes are listed in Table 1 which also reports on how many code applications were made for each topic, theme and subtheme, across how many publications.

**Table 1:** Final code set: Topics, themes, subthemes, code applications and publications covered.

Topic	Theme	Subtheme	Code applications	Publications covered
Intentions	Supporting Individual Self-Management		3	3
	Engagement with Data for Self-Reflection		6	4
	Supporting Patient Involvement		5	5
	Agency and Ownership		2	2
User Experience	Positive Experience	Instantaneous Access	4	3
		Facilitate Disclosure	3	2
		Longevity of Data	1	1
	Negative Experience	Fear of Misuse through Theft or Accidental Disclosure	5	4
		Fear of Judgment of Self-Management Practices	2	2
Design Features	Trust	Mutual Disclosure	2	2
		Host Credibility	5	3
		Personalisation (to appropriate user group)	4	3
	Identity	Anonymisation	2	1
	Privacy	Selective Data Sharing	10	8
		Informed Consent	3	2
		Privacy Policy	2	2
		Communication of Privacy Considerations	3	2
		Hiding Information	2	1
	Security	Data Handling Practices	4	3

We present our findings from this thematic analysis in Results, illustrated by quotes. Themes and sub-themes are shown in bold italics.

## 3 Results

**Fig 1:**
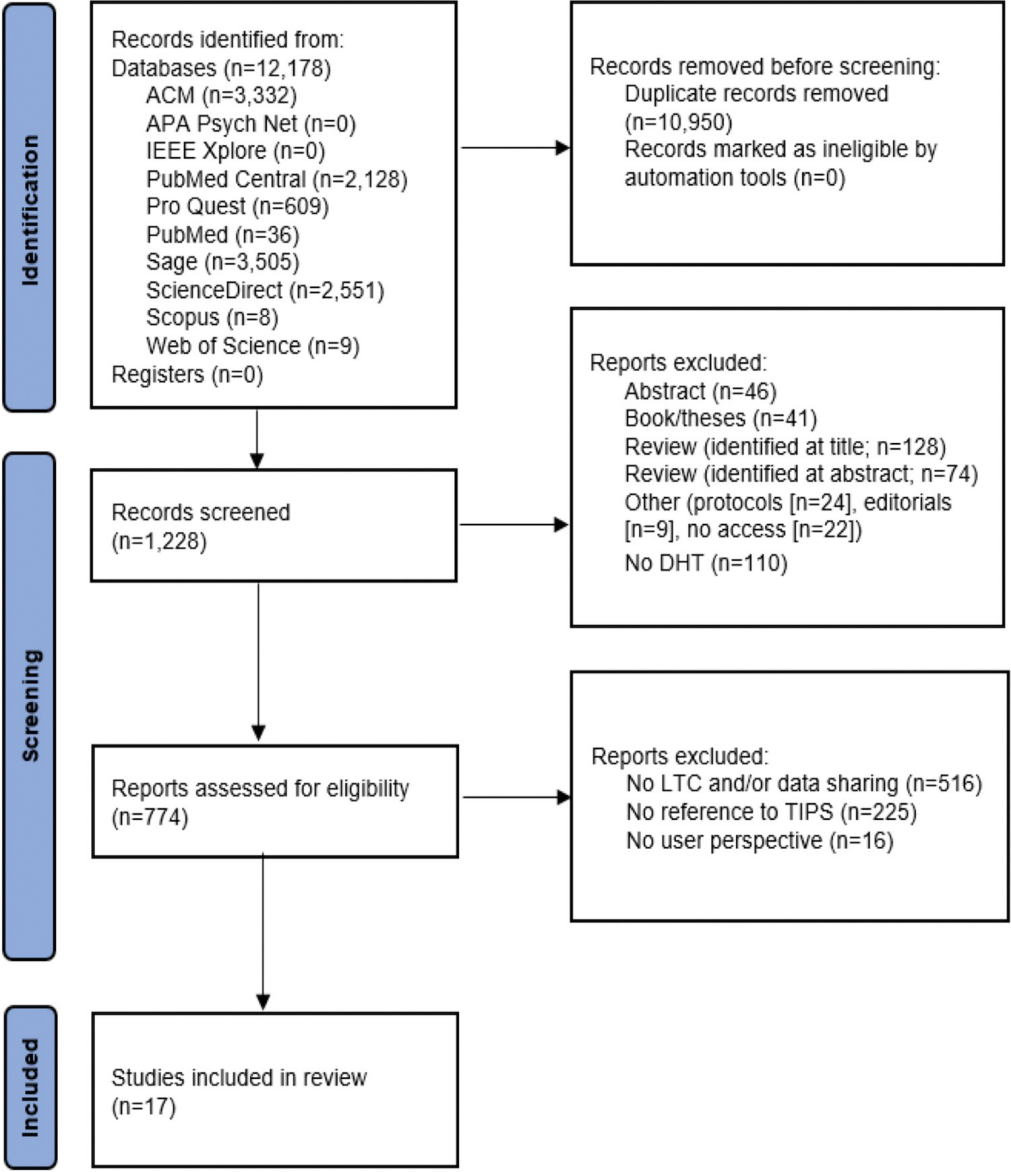
PRISMA flow diagram of study inclusion/exclusion. (DHT = digital health technology, LTC = Long Term Condition).

Through applying the selection strategy, we removed duplicates (n = 10,950), leaving 1,228 records remaining. Abstracts (n = 46) and books/theses (n = 41) were removed. Reviews identified by title read (n = 128) and by abstract read (n = 74) were removed. From the remaining 939 records, removals were made for ‘other’ reasons (e.g., protocols (n = 24), editorials (n = 9), no access (n = 22)). Records in which the abstract did not specify the inclusion of a digital health technology were excluded from the review (n = 110). The full text of the remaining 774 publications were reviewed, according to our selection strategy described earlier. From these, publications that did not include users with LTHCs who engaged in sharing their own personally generated health data with healthcare professionals or others were removed (n = 516). Publications with no reference to trust, identity, privacy or security (TIPS) of data sharing were removed (n = 225). For example, we excluded [34] because it contained no references to TIPS and we excluded [35] because there was no personally generated health data or involved people with LTHCs. While [36] deals with gathering and analysing personally generated health data, it does not focus on TIPS or the sharing of this data hence it was excluded. Finally, publications in which the reported data was not the direct perspective of the user (n = 16) were removed. Overall, there were 17 studies eligible for inclusion in the review, as presented in Fig 1.

### 3.1Characteristics of included publications

All of the included publications were collated into a table presenting their main characteristics (Table 2). The publications covered a range of LTHCs experienced by users but the most commonly investigated were mental health conditions such as anxiety and/or depression (n = 3), unspecified mental health issues (n = 1), alcohol dependency (n = 1), psychosis (n = 1) and early psychosis (n = 1), followed by cancer (n = 2), and diabetes (n = 2). Other LTHCs covered were spinal cord injury (n = 1), unspecified chronic disease (n = 1), Cystic Fibrosis (CF) (n = 1), Chronic Kidney Disease (CKD) (n = 1), Chronic Obstructive Pulmonary Disorder (COPD) (n = 1) and Inherited Retinal Disease (IRD) (n = 1). Across all of the publications reviewed, the studies included a total of 848 participants, with ages ranging from 4–75+ years. The majority of digital health technologies investigated in the publications were some form of web-based systems including platforms (n = 2), telemonitoring systems (n = 2), information systems (n = 1), ePHR prototypes (n = 1) or online portals (n = 1). However, we also note the rise of mHealth apps (n = 4). A large number of publications only investigated digital health technologies in general, as opposed to focusing on a specific one (n = 6).

**Table 2:** Included publications’ characteristics.

Ref	LTHCs	Sample Size	Age (Years)	Digital Health Technology	Study Type
Alexander et al. 2021 [37]	Chronic Obstructive Pulmonary Disorder (COPD)	17	61 (M) 44–75 (range)	TeleHealth program	Qualitative Focus groups
Allan et al. 2019 [38]	Psychosis (having experienced a relapse within the past two years)	21	Not reported	mHealth app	Qualitative Focus groups
Amann et al. 2020 [39]	Spinal cord injury	15	40.8 (M) 28–58 (range)	Web App	Qualitative Semi structured interviews
Bucci et al. 2018 [40]	Early psychosis (within the first three years of their initial episode	21	26 (M) 16–34 (range)	mHealth app	Qualitative Semi structured interviews
Cronin et al. 2018 [41]	Self-reported anxiety and/or depression	241	18–65+	Web app	Mixed Methods Questionnaire and open-ended feedback
Gilbert et al. 2022 [42]	Inherited retinal diseases (IRDs)	20	10–75	Web app	Qualitative Focus groups
Haggstrom and Carr 2022 [43]	Colorectal cancer survivors (having been diagnosed more than 12 month previous)	6	62.2 (M) 54–72 (range)	Web app	Qualitative Semi-structured interviews
Hall et al. 2021 [44]	Older adults with cancer (diagnosed in the past 18 months) with multi-morbidities (diagnosis of a LTHC)	14	73.8 (M) 67–85 (range)	Varied mHealth and web apps	Qualitative Semi-structured interviews
Nicholas et al. 2019 [45]	Anxiety and depression	211	38 (M) 8–66 (range)	mHealth app	Qualitative Questionnaire
Nightingale et al. 2017 [46]	Chronic Kidney Disease (CKD)	17	5–17	mHealth apps	Qualitative Semi-structured interviews and focus groups
Öberg et al. 2018 [47]	Type 2 Diabetes	11	65 (M) 50–78 (range)	Varied mHealth and web apps	Qualitative Semi structured interviews
Maria Pinas and Zanutto 2014 [48]	Paediatric diabetes	12	4–20	mHealth app	Qualitative Semi-structured interviews
Rutland et al. 2021 [49]	Cystic Fibrosis (CF)	5	17.7 (M) 15–20 (range)	mHealth app	Qualitative Semi-structured focus groups
Tieu et al. 2015 [50]	Chronic disease (none specified)	11	57 (M)	Web app	Qualitative Semi-structured interviews
You et al. 2019 [51]	Alcohol dependency	16	49.94 (M)	mHealth app	Qualitative Semi-structured interviews
Zhang et al. 2021 [52]	Mental health issues (within the past 12 months)	170	18–45 years	mHealth app	Quantitative Web-based survey
Zhou and Parmanto 2020 [53]	Mild to moderate depression	40	18–65 years	mHealth app	Mixed methods Questionnaire with follow-on interviews

Of the studies, 14 were solely qualitative and two utilised mixed methods using questionnaires coupled with opportunities for respondents to expand on their answers through an interview or open-ended questions. Only one study was quantitative. Due to the nature of the research area, most studies were exploratory in nature (n = 12).

**Fig 2:**
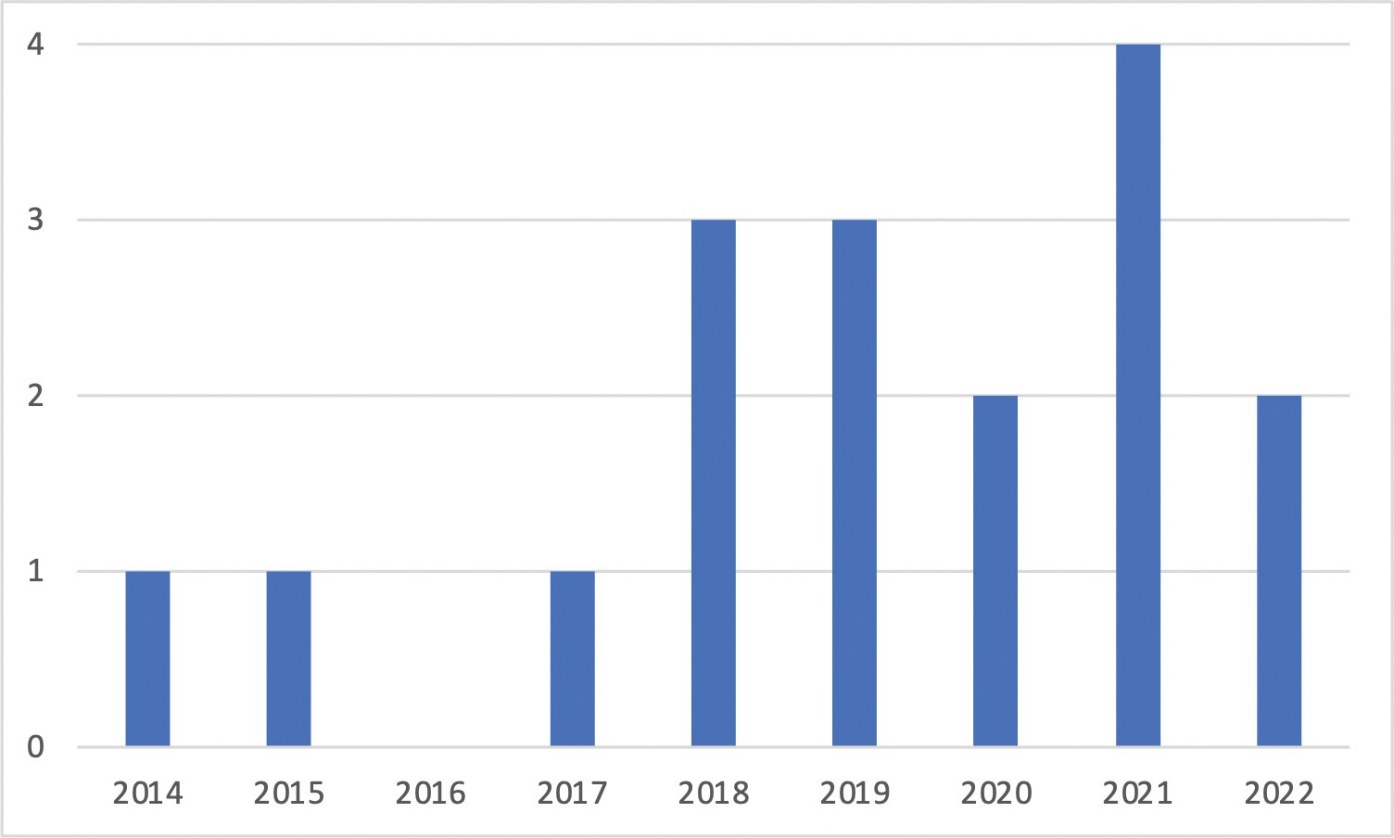
Number of relevant publications over years.

Looking over the years (Fig 2), we can see an increase of publications, and this is set to continue in 2022 for which we only had 2 months of data. It should be noted that there are no relevant publications before 2014, a full 6 years after the rise of apps.

### 3.2 Thematic analysis of included publications

#### 3.2.1 Intentions for using digital health technologies for tracking and sharing personally generated health data

We focused on reported intentions as they can be a motivating factor in adopting and using digital health technologies, distinct from user experience or TIPS considerations. We found four main themes arising from our analysis. These centre around supporting individual self-management, engagement with the data for self-reflection, supporting the patient-care provider relationship and agency and ownership. We discuss these themes in more detail next.

A common theme found in the reviewed work was ***Support Patient Involvement***, reported in five publications [41,45–47,51]. Users shared their personally generated health data to become more active in their personal health and care. Some positives noted from intention were: live updates of patient information; and treatment regimens and promoted adherence.

*“Participants from all groups suggested it would be beneficial if the app could be used to record information, including details of their treatment regimen (eg, medication, fluid target, and diet), appointments, and linkup with existing electronic patient records. This could promote treatment adherence by providing reminders and alarms, for example, for their medication and encourage children to record when treatment had been completed.” [46]*

Related to this theme is the sense of ***Agency and Ownership*** over their personally generated health data. Having control over their data was found to also go hand-in-hand with the ability to self-manage their condition:

*“Participants also described a sense of increased agency and being empowered to manage their conditions through a central place where they could self-administer changes in their personal details and be in control of their own health data, including how it was used in research.” [42]*

The sub-themes ***Engagement with Data for Self-Reflection*** [38–40,44] and ***Supporting Individual Self-Management*** [39,43,44] noted intentions that benefit individuals who share their data. After data is shared through an app, users are able to engage with their personally generated health data to reflect on symptoms and experiences of LTHCs. For example, a study with people with psychosis showed that digital health interventions with a symptom tracking functionality could “give people space to understand their experiences for themselves:

*“Participants were positive about the ability of smartphone technology to keep a track of their symptoms and experiences. Many thought that this ability would actually enhance their understanding of psychotic experiences. “I think it would be a great help because people would be able to see the warning signs very early on and go ‘hang on a second, this isn’t right, what do I need to do to help myself’” [40]*

Reflecting on one’s data is one way to ***Support their own self-management*** of a LTHC. Across three publications [39,43,44], participants highlighted the importance of tracking features in digital health technologies, in order for them to compare against others or to obtain further information about how their treatment is progressing. Others suggested that sharing to support self-management was beneficial for LTHCs so they could remain abreast of treatment efficacy.

*“One patient said*, *“It would be nice to be able to keep up with how your treatment’s going*, *kind of knowing if you’re… getting better… A lot of times when you have Cancer you always have a question mark over your head–where am I?”” [43]*

It was noted in three publications [38–40] that engaging with, and reflecting on, personal data promoted increased understanding of LTHCs and could further their self-management. This motivator to share seemed to endorse the concept of the ‘*informed patient*’ [54], allowing users to contemplate declines and improvements in their health and identify any causal factors.

#### 3.2.2 User experience

We analysed the experiences that users reported as part of sharing personally generated health data through digital health technologies. We divided these reports into two themes, positive and negative experiences, and we described their respective aspects which underlie these experiences as sub-themes.

**Positive experiences** were described in four of the 17 publications [39,40,42,47]. Three of these publications noted that data sharing within digital health technologies facilitated ***Instantaneous Access*** with relevant healthcare professionals, either as part of a routine engagement or when facing extraordinary circumstances e.g., away on holiday. It was suggested that users either felt more comfortable using a digital health technology as a medium to share data, as opposed to face to face interactions, or it was more convenient:

*“Technology was viewed as a good way of accessing help and support when needed because participants reported often feeling restricted by traditional face-to-face service provision. “It’s not like a GP [general practitioner] where you’ve gotta go up the road and then speak to him. [using technology] You can easily sit in your own home and read through the app…when I’m going to a GP…I’m silent.””[40]*

*“Individuals with SCI highlighted that the app could be particularly useful under extraordinary conditions (ie, outside of their daily routine). According to them, these were situations where even experienced wheelchair users might be hesitant or unable to use their usual means of contact (ie, telephone call). Frequently mentioned examples of such extraordinary circumstances were during holidays abroad, where one might not trust the foreign health care system, or when fallen ill. Wheelchair users also described nighttime and weekends as situations where the app could potentially come in handy. “You can directly get in touch with people, with specialists, I would appreciate that. For example, I once had a bruise when I lived in the south of France, then I actually did it exactly like that: I took pictures and thought about which doctors I knew and sent the photo.”” [39]*

Associated with this aspect is also that sharing personally generated health data through digital health technologies can ***Facilitate Disclosure*** which was described in two publications [39,40]. Users suggested that sharing personally generated health data via digital health technologies, as opposed to face to face, was advantageous if the data is perceived as sensitive which can then contribute to providing open and honest information:

*““Or you can also ask the question anonymously if you are embarrassed, if the pressure injury is in an embarrassing place, for example. [Then it’s probably easier to ask such a question], I could imagine. Maybe if you’re embarrassed about having to take a picture [of a pressure injury] on your butt.”” [39]*

*“The fact that an app is anonymous appealed to some because direct clinician contact can reinforce people’s sense of guilt or failure if they have not completed therapy tasks or complied with medication. “You don’t feel guilty if you haven’t done your homework.”” [40]*

One publication noted that users also experienced positive aspects of digital health technologies for the sharing of PGD in terms of ***Longevity of Data*** [42]. This was especially salient for those who had previous experience of such:

*“The following quotation from a patient with IRD (Inherited Retinal Disease) highlights an instance where she relocated to a different geographic area, which resulted in the loss of all her eye data, including her family history map of IRD. “From a personal perspective as well, we wouldn’t have lost our data and my mother would be able to know which side of the family all this came down from. So, if I’d have had that data we would still have that along with my letters from 1995. Yeah so, you will not lose your own data because it’s so important to you whereas we are just 1 in 60 million people in the United Kingdom.””[42]*

**Negative user experiences** were stated in five out of the 17 included publications [37,40,47,48,50]. The reported experiences fell into two broad themes, ***Fear of Misuse through Theft or Accidental Disclosure*** and ***Fear of Judgement of Self-Management Practices***.

Four publications dealt with the theme of ***fearing that shared data may be misused by others***. Concerns reported were theft or accidental disclosure:

*“Hackers getting [into] everything…I had to change banks because…they had everything—my name and address—my mom’s maiden name.”[50]*

*“If the app is asking you to pull it out every time you’re in a social situation, it gets embarrassing and that can add to the anxiety you feel in a social situation.”[40]*

The fear of accidental disclosure was especially present in rural communities, where there was a high chance that the user’s healthcare staff may possibly live within their local community and know the user and their family and/or friends personally. These negative experiences related to fear of misuse could be allayed by design features later discussed under Privacy and Security.

Users in two publications noted a ***Fear of Judgement of Self-Management Practices*** [47,48] when sharing personally generated health data through digital health technologies. While sharing data can also ***Facilitate Disclosure***, there is a fear that healthcare professionals might be critical of one’s self-management practices:

*“With the onset of diabetes, its “correct” management–as testified by the glycaemia levels and by the glycated haemoglobin value–is perceived as a factor used to evaluate the individual and his/her abilities, personal and parental, just as usually happens with educational performance. “There’s always this sentence that I will never forget: either you’re good or you’re not good! There’s a good patient, and there’s a bad one. So that being good means having good glycates. How can you say that a person is good on the basis of his or her glycates?”” [48]*

#### 3.2.3 Design features

We now describe the design features that seemed to positively influence TIPS, as extracted from the reviewed publications.

*Design features to inspire trust*. Two publications [43,46] reported that features to support ***Mutual Disclosure*** enhanced trust. Users from one publication recommended that digital health technologies provide a space to share qualitative personally generated health data with peers so that they may compare and contrast personal experiences. In the reviewed digital health technologies, the ability to mutually disclose personally generated health data with peers increased the user’s perceived value of the digital health technology, essentially promoting trust and prompting engagement via inclusivity.

Three publications [39,40,46] considered ***Host Credibility*** an important factor affecting user trust in digital health technologies. If data sharing through a digital health technology originated from what was deemed to be a credible host, user trust increased and concerns surrounding the trustworthiness were somewhat allayed:

*“Many participants said that endorsement of a DHI [Digital Health Intervention] by a valid institution (eg, university, health service, or respected, well known mental health charity) would be sufficiently reassuring and would increase DHI uptake.”[40]*

Some users believed that digital health technologies were better endorsed by individuals, as opposed to organisations, as they were less likely to be working toward an agenda they were unaware of. Recommendations from their care team were often sufficient for them to place trust in the digital health technology.

Sharing personally generated health data can be used for ***Personalisation***, as described in three publications [39,44,46], and this feature can then inspire trust and improve self-management. Instead of generic advice, users would like their shared data to be taken into account to shape advice according to their individual preferences, lifestyle and health.

*“Personalization technologies can help to better tailor the self-management app to an individual user’s needs and preferences […] by ensuring that recommendations regarding preventive measures are perceived as relevant and actionable rather than generic advice […] However, for personalization to be realised, users are required to disclose personal information.” [39]*

*Design features relating to Identity*. Design features relating to identity were rarely mentioned in the reviewed publications. Only one publication touched on this theme, exploring ***Anonymisation*** to protect patient’s personal identity in real life and their digital identity. While the benefits of sharing data, especially for advancing research into LTHCs, were clear, users felt more comfortable sharing their personally generated health data with third parties if their data had first been anonymized and that any personally identifiable data were removed from the shared data, with obvious connections to privacy and security:

*“Sharing of data in an anonymized form was perceived as a necessary safety feature by participants. “I would be happy to share my clinical information if it helps research or…but then maybe to disconnect my personal sort of data away from that.””[42]*

The same publication [42] described how their digital identity and associated data can contribute to a ***Larger Sample*** to build up information that is useful for the community, especially for rare diseases, which would also help healthcare professionals to increase understanding, education and inform optimum treatment pathway. This theme exemplifies benevolence as an aspect of trust but some users might value privacy over benevolence.

*Design features relating to privacy*. Privacy was the most prolific theme found in the included publications, with nine different publications covering design features that related to privacy. Eight publications [38–40,42,43,45,48,50] reported either user behaviour or requests for digital health technology that supported ***Selective Data Sharing***. Behaviour with digital health technology that does not support these design features often involved users censoring or withholding certain personally generated health data dependent upon the recipient—being *“a little less honest” [40]*. ‘Privacy by design’ [55] was suggested as an approach to support granular permissions to access and share data which in turn can increase trust. Users also highlighted that it would be beneficial to selectively grant permissions, for example, granting periods of access to shared data as opposed to unrestricted and the ability to remove permissions to certain parties if and when deemed appropriate. Related to this theme is ***Hiding Information***, referenced by one publication [53], to avoid accidental disclosure to bystanders who might observe app use—relating to the earlier Fear of Misuse through Theft or Accidental Disclosure theme. It is suggested that parts of apps could be hidden quickly and flexibly so that shared data cannot be seen by third parties.

‘Privacy by Design’ also covers ***Informed Consent*** in which users opt in to share data in the first place, underpinned by a ***Privacy Policy***, which four publications focused on [39,40,42,43]. Users want to have detailed information about who has access to the data and the purpose of accessing the data:

*“So, knowing, I suppose, where that data’s going, who’s got the data, or what it could be used for, the potential research…just knowing kind of an overview of what it’s for, what the reason this is for, then people can make that decision. I think it’s important.”[42]*

*“However, recent reviews of publicly available smartphone apps revealed that less than a quarter of those available for bipolar disorder included a privacy policy and less than 10% of those available for social anxiety provided organization information. This contrast between current information provided on public ally available mental health smartphone apps and the preference of service users for DHIs from trusted sources suggests that content information currently available may not be sufficient to alleviate service user privacy concerns, thus potentially negatively impacting engagement. Future developers must ensure that clear and explicit statements regarding privacy and organizational sources are made available.”[40]*

Informed consent and the availability of a privacy policy highlights the importance of transparency of data sharing, facilitating a sense of ownership of said data, the credibility of the host, and trust in and engagement with the digital health technology. This informed consent also extends to re-use of the data for purposes other than originally intended by the digital health technology or by organisations not originally part of the informed consent. A problematic aspect is that if data were shared anonymously, there should be no way that individuals can be traced–informed consent thus needs to be given to *any* organisation for *any* purpose right from the start. Indeed, even if the data is not anonymised, General Data Protection Regulation (GDPR) requires clarity around data controllers, data processors, and the purpose for which the data is stored and processed.

Whilst the information is found in privacy policies, our analysis suggests that data use, permissions and users rights regarding their data are not always clear, concise and transparent, as they are evidencing the need for reiteration. ***Communication of Privacy Considerations*** that digital health technology were making had an impact on privacy concerns [39,42]. To ensure the transparency of the privacy statement, developers must ensure that they relay information in a clear, concise, accessible method:

*“All this information needs to be accessible to prospective users in a clear and concise format, informing them about their rights to access and control their data. However, how personal information is handled by mobile health apps often remains unclear. Some apps, for example, track and share user data continuously by default, even when the app is not in active use, sometimes without explicitly informing users about these tracking practices in their terms of use.” [39]*

This may be achieved in various ways, from visualisation techniques to comprehensive privacy information. Communicating privacy policies well can lead to a sense of ownership of data, and in turn also inspire trust.

*Design features relating to Security*. Security design features were covered by three different publications [39,40,42]. In all these publications, we identified the theme of ***Data Handling Practices***, which were important to users to help allay their Fear of Misuse by Others. Users were keen to know how their data was protected, and the host organisation’s data governance:

*“In particular, participants raised questions regarding the storage, sharing, backup, and deletion of personal data. “On the one hand, that would be an advantage for me and certainly also an advantage if you could read it again. On the other hand, I simply have to ask myself, is the data even stored safely? Does it really stay where it is or is it passed on to third parties?””[39]*

*“About two-thirds (16/21, 76%) of participants expressed concerns about data protection and information governance. However, participants stated that their fears about information safety could be allayed if services reassured them about data safety.” [40]*

### 3.3 Summary of results

**Table:** 

**Research gap**	**Results**
Evidence for designing digital health technologies that support sharing of personally generated health data with others	17 publications that empirically investigate use of these technologies, increasing number of publications in recent years
Lack of systematic and quantifiable measures in studies in this area	14 qualitative, 2 mixed-methods, 1 quantitative study
Intentions for sharing personally generated health data with others, motivating adoption and use of digital health technologies	Sharing data supports individual self-management and the patient-care provider relationship, engagement with the data for self-reflection, and provides a sense of agency and ownership
Positive user experiences	Facilitating instantaneous access to healthcare professionals, facilitating disclosure of sensitive data, and longevity of data.
Negative user experiences	Fear of misuse and fear of judgement of self-management practices.
Design features to inspire trust	Mutual disclosure, host credibility, personalisation
Design features relating to identity	Anonymisation and contribution to larger sample
Design features relating to privacy	Selective data sharing, hiding information to avoid accidental disclosure, presence of informed consent and privacy policy, clear communication of privacy considerations
Design Features relating to security	Communication of robust data handling practices

## 4 Discussion

Our review adds to the continuing and evolving body of work that investigates digital health technology in the self-management of patient’s conditions [1–3,11,12]. While some of the research has started to investigate ePHRs, we focussed our review on digital health technology that supports sharing personally generated health data with others, which, to date, is an area that has not received much attention.

Our review of published works in the digital health technology space that directly report on and address TIPS in their designs has yielded only a small set of papers. Given the plethora of digital health technologies that are being developed based on personally generated health data and that these data may need to be shared, it is surprising that there are so few publications that investigate this topic. While there are more and more systems being designed that allow patients to share data with others–healthcare professionals, other patients, or even other organisations–through digital health technology platforms, the evidence base for making design choices during systems development is relatively poor. While there is increasing attention given to this topic, we argue that there needs to be more empirically backed research on how to design digital health technologies that support the sharing of personally generated health data so that we can develop successful, evidenced-based self-management solutions.

We do encourage researchers and practitioners to contribute further to knowledge in this area, and report in detail how these technologies were implemented, how interfaces were presented to users, and more importantly the effects that these design choices had on system use. This necessitates the adoption of common and standardised metrics to assess the user experience, and especially the effects on trust, privacy and security. For example, quantitative instruments to measure the user experience, such as SUS [56], UEQ [57] or meCue [58], are becoming more common outside of digital health technologies, with the added benefit that it is then possible to benchmark systems against a standard. Questionnaires to measure TIPS are also being developed, such as the trust scales [59] and Privacy Comfort Scale [60]. As it stands, in the absence of quantitative measures, it is impossible to conduct any rigorous systematic reviews to evidence and inform the choice of digital health technology designs. It is hoped that in the future more quantitative work, especially around usability and TIPS, will enable a systematic review to be carried out.

Yet our reflexive thematic analysis has uncovered interesting directions to explore when developing digital health technologies that support data sharing. When we investigated the intentions behind sharing data with others, we found that tracking/sharing personally generated health data can be beneficial for different stakeholder groups in order to support their self-management, however, it is worth noting the ’self’ oriented nature of the sharing intentions: sharing data is focused on their own benefit, rather than sharing to help others in some way. Thus, data sharing with others almost becomes a by-product rather than the main functionality. This is also supported by previous research [61] into barriers to sharing data which supports that data is shared overwhelmingly for better management of their own condition instead of improving the health of others. It is hence necessary to consider how to motivate users to keep sharing data and how data sharing can be used for an individual’s benefit. This insight warrants further research into how shared data can be used by others to provide better self-management, for example, for better engagement with and access to healthcare professionals, or how shared data in combination with their own can help an individual to make better, more informed choices. This also suggests a lack of design exploration in this space, which supports users to interpret shared data in relation to their own.

Other noteworthy tensions arise also when we explored the design features around privacy and their intentions. A number of reports we reviewed stressed the importance of selective data sharing which places autonomy into the hands of the user. As previous work has pointed out [62], these desiderata are often in conflict with principles eschewed in biomedical ethics, such as equality and beneficence, and also lower the benevolence towards others with whom data is shared.

Many design features related to privacy and security and suggested how to increase user experiences around these themes. We were unable to establish how these design features, and privacy and security in general, related to trust in digital health technologies and how they might interact to increase or decrease trust. We will have to await further studies to provide knowledge in this area.

## 5 Conclusions

We have reported a scoping review of literature between 2008 and 2022 on the design of digital health technologies for sharing data with others, and the implications for intentions, user experience and TIPS. Conducting a thematic analysis of the 17 publications yielded the following insights:

- Sharing data was seen as a way to improve self-management of conditions by individuals, however, sharing behaviour was self-centric rather than focused on benevolence to others.
- While there seem to be positive aspects of instantaneous access and benevolence to sharing data, many fear misuse through Theft or Accidental Disclosure. Sharing data can also give rise to fears of being judged about their Self-Management Practices. This might place barriers in terms of privacy, security and indeed trust.
- Design features relating to trust were focused on host credibility and personalisation. It was found that mutual disclosure of sharing data can inspire trust.
- Identity was not a strong theme in our analysis and mainly focused on anonymisation of data. This might be due to lack of digital health technologies that focused on sharing with peers, where anonymisation matters more.
- Security was discussed relatively little and only in terms of high-level mechanisms. Little is still understood about security for sharing data in digital health technologies that is specifically user-focused.
- Privacy was the most covered aspect in terms of design features. Particularly important to users were the ability to selectively share data with others, and issues of transparent and informed consent to sharing data to allay their privacy concerns. Addressing these issues might lead to increased trust in and engagement with digital health technologies.

This area of work is still in its infancy, and it arguably requires interdisciplinarity to engage with, given the intersection of health and care with technology and design. We call upon researchers and practitioners working in Digital Health, HCI, Health Security, and related fields, to report their experiences with digital health technologies which support data sharing and the collection of more robust measures that can yield insights into the design of future technologies that can improve the self-management of chronic conditions.

## Supporting information

S1 Appendix PRISMA-ScR checklist. (PDF)
